# Two major genes associated with autoimmune arthritis, *Ncf1* and *Fcgr2b*, additively protect mice by strengthening T cell tolerance

**DOI:** 10.1007/s00018-022-04501-0

**Published:** 2022-08-14

**Authors:** Qijing Li, Jianghong Zhong, Huqiao Luo, Vilma Urbonaviciute, Zhongwei Xu, Chang He, Rikard Holmdahl

**Affiliations:** 1 The Second Affiliated Hospital of Xi’an Jiaotong University (Xibei Hospital), Xi’an, China; 2 Medical Inflammation Research, Department of Medical Biochemistry and Biophysics, Karolinska Institute, Stockholm, Sweden; 3 Department of Hematology, the First Affiliated Hospital of Xi’an Jiaotong University, Xi’an, China; 4 School of Engineering Medicine, Beihang University, Beijing, China; 5 Department of Cardiovascular Medicine, The First Affiliated Hospital of Xi’an Jiaotong University, Xi’an, China

**Keywords:** Fc receptor, p47phox, NOX2, Type II collagen, Immune regulation, Rheumatoid arthritis

## Abstract

A breach of T cell tolerance is considered as a major step in the pathogenesis of rheumatoid arthritis. In collagen-induced arthritis (CIA) model, immunization with type II collagen (COL2) leads to arthritis in mice through T cells responding to the immunodominant COL2_259–273_ peptide. T cells could escape from thymus negative selection because endogenous COL2_259–273_ peptide only weakly binds to the major histocompatibility complex class II (MHCII) molecule A^q^. To investigate the regulation of T cell tolerance, we used a new mouse strain BQ*.Col2*^*266E*^ with homozygous D266E mutations in the *Col2* gene leading to a replacement of the endogenous aspartic acid (D) to glutamic acid (E) at position 266 of the COL2_259–273_ peptide, resulting in stronger binding to A^q^. We also established BQ*.Col2*^*264R*^ mice carrying an additional K264R mutation changed the lysine (K) at position 264 to eliminate the major TCR recognition site. The BQ*.Col2*^*266E*^ mice were fully resistant to CIA, while the BQ*.Col2*^*264R*^ mice developed severe arthritis. Furthermore, we studied two of the most important non-MHCII genes associated with CIA, i.e., *Ncf1* and *Fcgr2b.* Deficiency of either gene induced arthritis in BQ*.Col2*^*266E*^ mice, and the downstream effects differ as *Ncf1* deficiency reduced Tregs and was likely to decrease expression of autoimmune regulator (AIRE) while *Fcgr2b* did not. In conclusion, the new human-mimicking mouse model has strong T cell tolerance to COL2, which can be broken by deficiency of *Fcgr2b* or *Ncf1*, allowing activation of autoreactive T cells and development of arthritis.

**Supplementary Information:**

The online version contains supplementary material available at 10.1007/s00018-022-04501-0.

## Introduction

Rheumatoid arthritis (RA) is a chronic autoimmune disease which is believed to be initiated and driven by a breach of T cell tolerance to unknown self-antigens [1]. RA is classified by a set of clinical criteria [2], the major group, classified as seropositive RA, is characterized by increased levels of certain autoantibodies like rheumatoid factors (RF) and antibodies to citrullinated proteins (ACPA) which could appear several years ago before the clinical onset [3]. Seropositive RA is strongly associated with a related set of major histocompatibility complex class II (MHCII) alleles [4, 5], but the association is not linked to the appearance of autoantibodies but rather to the onset of an inflammatory attack on the joints [6]. MHCII molecules are receptors on antigen-presenting cells (APCs), which bind and present antigenic peptides to T cells. The peptides presented to T cells prior to the onset of RA are believed to be derived from self-proteins, but the regulative factors operating in this process remain to be explored. Clues are sought in attempts to identify variant genes outside the MHCII region, but those have been difficult to identify and to study functionally due to linkage disequilibrium. However, qualified guesses indicate several genes that may control T cell tolerance, including Fc gamma receptor (FCGR) locus containing FCGR2B polymorphism and Neutrophil cytosolic factor 1 (NCF1) polymorphism which was identified in rats [7] and later been positioned both as a copy number variation (CNV) and disease causative SNP in human [8–10].

To be able to understand how disruption of T cell tolerance could lead to the development of arthritis, we need to study suitable animal models. Since RA is a highly heterogeneous disease related to largely unknown genetic and environmental factors, there are variant animal models developed and used. The most thoroughly characterized mouse model is collagen-induced arthritis (CIA) which is commonly induced by immunization with non-self (bovine/rat/chicken/human) COL2 [11]. It is also inducible with mouse COL2 but then results in a less susceptible but more chronic disease [12]. However, in all cases, the immune response is entirely dependent on T cell recognition of a single peptide located at position 259–273 number from the start of the COL2 triple helical structure which binds to the MHC II molecule A^q^[13]. In this immunodominant peptide, the main TCR interaction site is a lysine at position 264 (K264), which can be hydroxylated and glycosylated [14]. Also, mouse COL2 differs from the non-self COL2 at position 266 by only one aspartic acid (D266). The non-self COL2 instead has glutamic acid (E266) leading to 12 times tighter binding to A^q^, which explains why it is more efficiently presented and elicits a stronger response after immunization [15, 16].

To investigate the mechanism of T cell tolerance operating in CIA model, we have previously developed a mutated COL2 (D266E) transgenic mouse strain, i.e., the mutated mouse collagen (MMC) strain to allow the non-self COL2 to be expressed in the joint cartilage [17]. MMC mice were less susceptible but not completely resistant to CIA, depending on the interaction of non-MHC genes. Using MMC mice, we showed that autoreactive T cells specific for the non-glycosylated COL2_259–273_ peptide undergo efficient central tolerance in an autoimmune regulator (AIRE) dependent manner [18]. However, the MMC transgenic strain still expresses the endogenous mouse COL2 which likely leads to the formation of heterotrimers in the cartilage and confounding immune responses. To establish a more physiologic context for the regulation of T cell tolerance, we generated a new strain BQ*.Col2*^*266E*^, with homozygous D266E mutations, leading to a complete replacement of aspartic acid (D) with glutamic acid (E) at position 266 in the immunodominant COL2_259–273_ peptide. In addition, we also made the BQ*.Col2*^*264R*^ strain which carries K264R mutations besides existing D266E mutations, leading to a change in TCR recognition site by replacing lysine (K) with arginine (R) at position 264, which has proved to prevent T cell recognition of the COL2 peptide [19]. After immunization with non-self COL2, no arthritis was observed in BQ*.Col2*^*266E*^ mice, while BQ*.Col2*^*264R*^ mice developed severe arthritis. Also, we have previously been able to position several genetic polymorphisms controlling autoimmune arthritis in mice and rats outside the MHC region—*Ncf1* controlling oxidative burst as one of the subunits of NOX2 complex [20, 21] and the inhibitory Fc receptor *Fcgr2b*[22]. To study whether they could break T cell tolerance and allow the development of CIA driven by autoreactive T cells, we intercrossed *Fcgr2b* and *Ncf1* deficient mice with the BQ*.Col2*^*266E*^ strain and found that both genes could additively break the T cell tolerance and allow the development of arthritis.

## Materials and methods

### Mice

Mice were bred and kept at Comparative Medicine’s Annex (KM-A) which is an animal facility of barrier C in Karolinska Institute in Stockholm, Sweden. Mice were housed under specific pathogenic-free conditions (FELASA II) in intra-ventilated cages with sterilized wood shavings and paper nesting material, fed with standard chow and water ad libitum. The animal genotyping and experimental protocols were approved by Stockholm regional animal ethics committee, Sweden (12923–2018, N35/16). Experiments using arthritis models were performed in male mice that were between 10 and 12 weeks old. For experiments with naïve mice, mice between 8 and 10 weeks old of the same sex were used. All the mice with BQ*.Col2*^*266E*^ background are littermates.

B10.Q (C57BL/10.Q) founders have been fully backcrossed and maintained in our laboratory for a long time. *Fcgr2b* knockout (*Fcgr2b*^*−/−*^*)* mice and *Ncf1* mutated (*Ncf1*^*m1j/m1j*^) mice were obtained from Jackson Laboratory and were fully backcrossed into B10.Q background for more than 10 generations in our laboratory to introduce H2-q haplotype in the MHC locus. The knock-in C57BL/6 J.*Col2*^*D266E*^ (BQ*.Col2*^*266E*^) and C57BL/6 J*.Col2*^*K264R*^ (BQ*.Col2*^*264R*^) mouse lines were generated by introducing point mutations at exon22 (p.D466E, c.1398 T > A) or (p.K464R, c.1391A > G; p.D466E, c.1398 T > A) of Collagen Type II Alpha 1 Chain (*Col2a1*) gene via CRISPR/Cas9 technology, respectively (Shanghai Biomodel Organism Science and Technology Development Co., Ltd, China). In our animal facility, these lines were backcrossed at least 5 generations into *B10.Q* background to introduce the H-2q haplotype in the MHC locus. B10Q*.Ncf1*^*m1j/m1j*^ and B10Q*.Fcgr2b*^−/−^ were crossed with BQ*.Col2*^*266E*^ homozygous mice to produce BQ*.Col2*^*266E*^*.Ncf1*^*m1j/m1j*^ and BQ*.Col2*^*266E*^*.Fcgr2b*^*−/−*^ mice with their littermates.

### Animal models

Three kinds of RA mouse models were used in this study. Collagen-induced arthritis (CIA): mice were immunized intradermally at the base of the tail with 100 ul emulsion made from 100 µg rat or bovine type II collagen (COL2) produced in our laboratory dissolved in acetic acid solution and complete Freund’s adjuvant (BD, Difco) in one-to-one configuration mixed by an emulsifier machine [23] (BTB, POWER-Kit). Mice were boosted at day 35 with 50 ul emulsion made of 50 µg COL2 in incomplete Freund’s adjuvant (BD, Difco) mixed by machine. The scoring usually started at around day 15, with an interval of 2–3 days. Glucose-6-phosphate isomerase-induced arthritis (GIA): mice were immunized intradermally at the base of the tail with 100 ul emulsion made from 10 ug human GPI_325–339_ (Biomatik) in Dulbecco’s phosphate-buffered saline (DPBS) (Thermo Fisher Scientific, Gibco) and CFA (BD, Difco) in one-to-one configuration. The scoring started from day 8 and was recorded every day. Collagen antibody-induced arthritis (CAIA): pathogenic anti-COL2 antibodies (M2139, ACC1, 15A, L10D9) were produced by corresponding hybridomas and then purified based on affinity chromatography [24]. After purification, antibodies were dialyzed in PBS and then concentrated to 10 mg/ml. These 4 antibodies were mixed at same amount and stored at 4 ℃ or on ice prior to injection. All mice were injected with 2 mg (200 ul) of cocktail on day 0, intravenously. The scoring started right after the antibody cocktail injection and was recorded for 3 weeks with an interval of 1–2 days. The scoring method was same for all these models. Each visibly inflamed (erythema and swelling) ankle or wrist was given up to 5 points, each inflamed knuckle and toe was given 1 point, together the maximum score per mouse was 60. Mice were killed when reach the experiment endpoint or ethics endpoint according to the ethical permit.

### ELISA

For measurement of anti-COL2 antibody, blood was collected from mice on day 21, 35 and 49. Serum was obtained from blood after 10,000 rpm centrifugation for 10 min and kept in − 20 freezers until used. The 96-well ELISA plates (Thermo Fisher Scientific, Nunc MaxiSorp) were coated with COL2 diluted in DPBS to a final concentration of 10 µg/ml, 50 ul per well, overnight at 4 °C. After coating, plates were washed 5 times with ELISA buffer (0.1% Tween 20 in PBS). Plates were then blocked with 1% BSA in DPBS for 1 h at RT. Serum was diluted with DPBS into different concentrations: 1:1600 for IgG_1_ detection, 1:400 for IgG_2b_ detection, 1:6400 for total IgG detection. The plates were washed 5 times and 50 ul of the diluted serum was added per well in triplicates, then incubated for 2 h at RT. The plates were washed 5 times and the detective antibody was added (1:4000 in DPBS): goat anti-mouse IgG(H + L)-HRP (Southern Biotech), goat anti-mouse IgG1-HRP (Southern Biotech) and goat anti-mouse IgG2b-HRP (Southern Biotech), 50 ul per well, incubated for 1 h at RT. The plates were washed 5 times, 50 ul of 1:5000 ExtrAvidin^®^ -Peroxidase (Sigma) DPBS dilution was added per well and incubated for 45 min at RT. The ABTS solution was prepared by adding 1 ABTS tablets (Boehringer Mannheim) into 5 ml of 1X ABTS buffer diluted from 10X ABTS buffer (Boehringer Mannheim) for 1 plate. The plates were incubated in the dark for around 20 min until the color appears then read at 405 nm (OD_405_). For quantification of total IgG, a standard serum sample was used allowing comparison with a standard curve. For IL-2 detection, the experimental procedure was essentially the same. Plates were coated with homemade IL-2 monoclonal antibodies (Clone: JES6-IA12) diluted with DPBS to a final concentration of 2 ug/ml. After 5 times washing, 50 ul of medium containing IL-2 was added per well and the plates were incubated for 2 h at RT or overnight at 4 °C. The secondary biotinylated antibody (Clone: JES6-5H4) was diluted to 0.5 ug/ml. After washing, 50 ul of Eu-labeled streptavidin diluted with DPBS (PerkinElmer, 1:1000) were added per well and the plates were incubated for 30 min. After washing, the enhancement solution (PerkinElmer) was added, and the plates were read at 620 nm (OD_620_).

### Histology

Mice were killed on day 90 of the CIA experiment. The left hind paw was collected and the skin around the joint was removed. Paws were fixed in 4% formaldehyde solution (Histolab), then decalcified with 10% EDTA for over 3 weeks. Dehydration was done by a machine in Histological Core Facility of KI. Tissue was embedded in paraffin and cut into 5 μm slides (Microtomes HM360). All the slides were stained with hematoxylin–eosin (H&E).

### Flow cytometry

Inguinal lymph nodes were collected 10 days post immunization; thymi were collected from naïve mice. To make single-cell suspensions, lymph nodes were directly ground up and passed through 40 µm filters, while thymi were cut into pieces and digested with enzyme solutions as previously described [25]. Between every step below the samples were washed with DPBS 150–180 ul and were centrifuged at 350*g* for 5 min at RT to remove supernatant. Cells were added into 96-well U bottom plates at 1–10 × 10^6^ cells per well (adjusted for certain experiments), then stained with LIVE/DEAD™ Fixable Dead Cell Stain (Thermo Fisher Scientific, Invitrogen) for 5 min on ice. Samples were blocked with homemade FcR block (Anti-mouse CD16/CD32, Clone: 2.4G2) for 10 min on ice, then stained with the antibodies (listed below) in 50 µl of PBS dilution on ice for 20 min in the dark. Cells were then washed with PBS and FACS buffer and ran on the Attune NxT flow cytometer (Thermo Fisher Scientific, Invitrogen). For intracellular staining, cells were washed, fixed, and permeabilized using BD Cytofix/Cytoperm (BD) set and then stained intracellularly with antibodies diluted with permeabilization buffer (BD) all according to manufacturer’s instructions. For FOXP3 staining, samples were fixed and permeabilized by eBioscience Foxp3/Transcription Factor Staining Buffer (Thermo Fisher Scientific, Invitrogen) set according to the protocols. For intracellular cytokine staining, cells were stimulated in vitro with the mixture of phorbol 12-myristate 13-acetate (PMA) 100 ng/ml, ionomycin 1 µg/m1, and BFA 10 µg/ml in 100 ul medium per well for 4 h at 37 °C prior to fixation, permeabilization, and staining.

#### Antibodies and others

Antibodies: antibodies were purchased from BD, Biolegend, Thermo Fisher Scientific unless stated otherwise. CD16/CD32 (2.4G2, in house); CD3 (145-2C11); TCRb (H57-597); TCRgd (GL3); Gr-1 (RB6-8C5); Ter-119 (TER-119); CD4 (RM4-5); CD8 (53–6.7); H-2, I-Aq (M5/114.15.2); F4/80 (BM8); CD19 (1D3, 6D5); CD11b (M1/70); Ly6G (1A8); Ly6C (HK1.4); CD11c (HL3, N418); FOXP3 (FJK-16s); CD25 (7D4); CD44 (IM7); CD69 (H1.2F3); CD45 (30-F11); EpCAM (G8.8); IL-33R (U29-93); IFN-γ (XMG1.2); IL-17A (TC11-18H10.1); NK1.1 (PK136); AIRE (5H12); NCF1(D-10); Ly51 (6C3); CD32b (AT130-2). Others: UEA-1, FITC conjugated (Thermo Fisher Scientific, Invitrogen).

### Oxidative burst detection

For Fc receptor-induced ROS detection, spleens were isolated and ground through 40 ul filters to make single-cell suspensions. Red cells were lysed with ammonium-chloride-potassium (ACK) buffer. Cells were stained for cell markers and then stimulated by Fc OxyBURST™ Green Assay Reagent (Thermo Fisher Scientific) which are IgG-bound immunocomplex conjugated with H_2_DCF according to the protocols from the manufacturer. Samples were then washed with cold DPBS and put on the ice for 10 min to stop the ROS production then ran on a flow cytometer at relatively high speed.

### ELISpot

The 96-well ELISpot plates (Millipore) were prewetted in an ultra clean bench with 15 ul freshly prepared 35% EtOH for less than 1 min, washed with sterilized DPBS and coated with anti-mouse IFN-γ capture antibody (Clone: R46A2, Mabtech) diluted to 10 ug/ml, 50 ul per well, then incubated overnight at 4 °C. Single cell suspensions from lymph nodes were prepared as described in the Flow cytometry part. The plates were washed 2 times, then non-modified or galactosylated COL2 peptide was added, cells were diluted with complete DMEM (DMEM + Glutamax (Gibco), 5% FBS (Gibco), 60 µg/ml penicillin C (Sigma)) 200 ul in total per well to eventually have 0.5 M or 1 M cells and peptides at a final concentration of 10 µg/ml. Concanavalin A (diluted with complete DMEM,1:1000) was used as positive control, medium without any stimulator was used as negative control. The plates were incubated for 24 h in a cell incubator, the liquid was flicked out, whereafter the plates were washed twice with DPBS and 3 times with ELISpot buffer (0.01% Tween 20 in PBS). Biotinylated anti-IFN-γ antibodies (clone: An18, Mabtech) were added at 50 ul per well, 4 ug/ml diluted with 0.5% BSA/DPBS, then incubated for 2 h at RT. The plates were washed 5 times with ELISpot buffer, then 50 ul of streptavidin–alkaline phosphatase (1:2500 in DPBS) were added and incubated for 30–45 min at RT. The plates were washed with buffer and DPBS 3 times, respectively, and 100 ul pre-filtered (0.45 µm) BCIP (Sigma) solution (1 tablet in 10 ml ddH_2_O) was added, and plates were incubated in the dark until the spots appeared. The plates were thoroughly rinsed with water, dried, and counted using ImmunoScan ELISpot Analyzer (CTL Europe).

### Tetramer staining

The biotinylated Gal-COL2_259–273_-specific tetramers were previously produced and stocked at − 20 °C. The single cell suspensions from lymph nodes were prepared in cDMEM. Then 5 M cells were added per well in 96-well U bottom plates, the plates were then centrifuged, and supernatant was discarded. The cells were resuspended with 200 ul cDMEM containing 50 nM Dasatinib (SantaCruz) and homemade FcR block (Anti-mouse CD16/CD32, Clone: 2.4G2) and incubated at RT for 30 min. The plates were centrifuged, and supernatant was discarded, then resuspended with 50 ul biotinylated Gal-COL2_259–273_-specific tetramers and streptavidin PE conjugate (Thermo Fisher Scientific, Invitrogen) mixed in ddH2O (final concentration of tetramers around 0.4 mg/ml), the plates were incubated in the dark at RT for 30 min. 10 ul antibody mixture were added per well and plates were incubated for another 30 min. The cells were washed with FACS buffer twice and then analyzed by flow cytometry.

### Antigen-presenting assay

HCQ3 and HCQ4 T cell hybridomas have previously been described [26]. These cells were thawed and cultured in cDMEM overnight. Thymi were isolated and ground into single-cell suspensions as described above. Thymic DCs were sorted using CD11c MicroBeads UltraPure (Miltenyi biotec) and MS column (Miltenyi biotec) according to the manufacturer’s protocol. Subsequently, 0.05 M of DCs and 0.15 M HCQ3 or HCQ4 cells with antigens (COL2 molecule, final concentration 100 ug/ml) in cDMEM or pure medium were added per well in 96-well U bottom plates and incubated for 15 h at 37 °C. 5 mM *N*-*acetyl*-*L*-*cysteine* (Nac) were added in some groups to remove ROS. Plates were centrifuged at 350*g* for 10 min, 50 ul of supernatant were taken for IL-2 detection (described in ELISA part), the rest were resuspended, stained, and analyzed by flow cytometry.

### Statistics

Statistical analyses were performed with GraphPad Prism software, version 8.0.1 (GraphPad Software, San Diego, CA). Unless otherwise stated, the Mann–Whitney *U* test was used to compare the means of two groups. Data were shown as mean ± standard error or mean ± standard deviation. *P* value < 0.05 was considered as significant: *p < 0.05, **p < 0.01, ***p < 0.001, ****p < 0.0001.

## Results

### BQ***.Col2***^***266E***^ mice have strong T cell tolerance reducing arthritis susceptibility, whereas BQ***.Col2***^***264R***^ mice develop severe arthritis

**Fig. 1:**
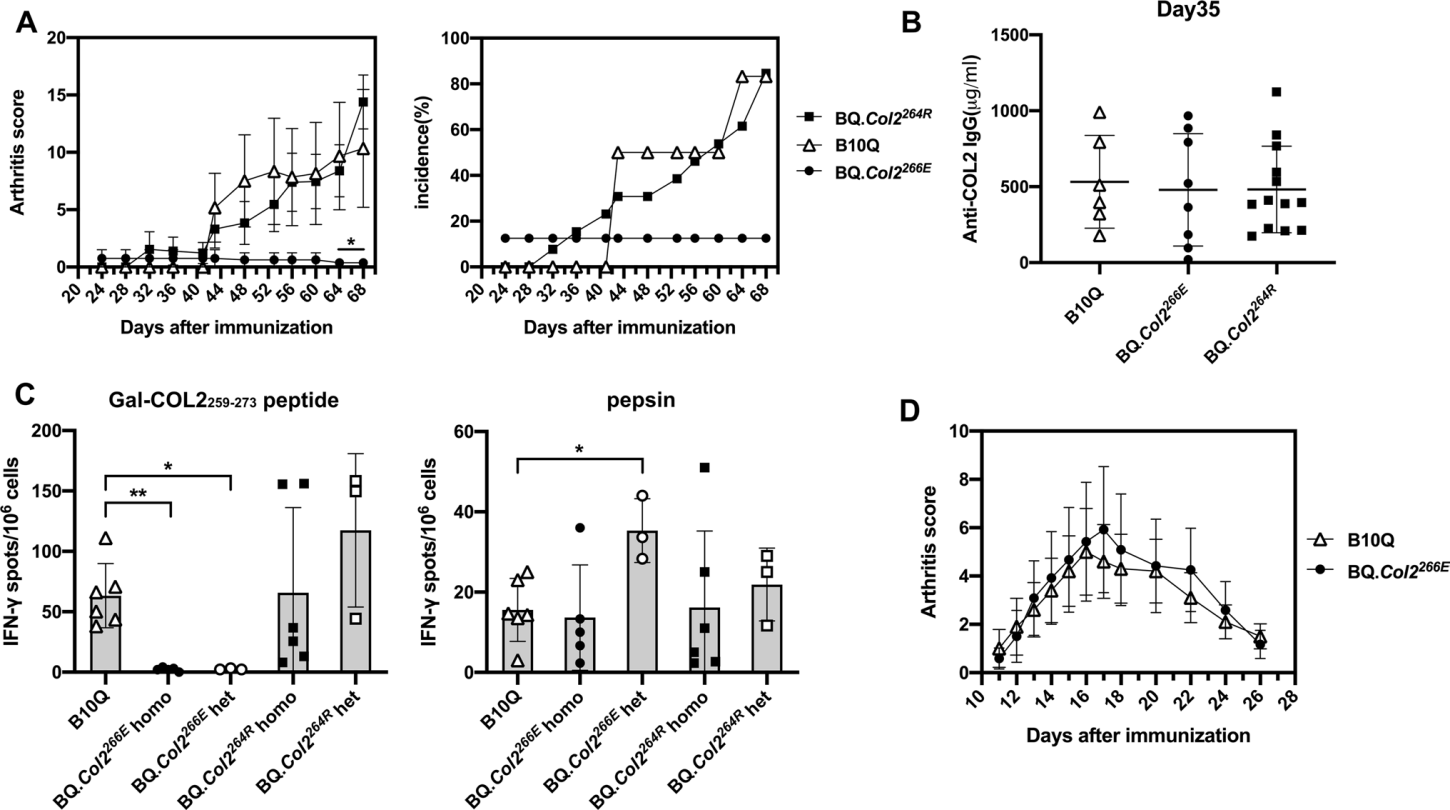
BQ*.Col2*^*266E*^ mice are resistant to CIA whereas the BQ*.Col2*^*264R*^ mice develop severe arthritis. BQ*.Col2*^*266E*^ mice carried homozygous D266E mutations in *Col2* gene thus expressing non-self COL2. BQ*.Col2*^*264R*^ mice had an additional K264R mutation to eliminate the TCR recognition site of the immunodominant peptide of COL2. **a** CIA was induced at day 0 by i.d. injection of rat COL2 emulsified in CFA (Male mice, BQ*.Col2*^*266E*^: *n* = 8, BQ*.Col2*^*264R*^: *n* = 13, B10Q: *n* = 6) and mice were boosted at day 35 with rat COL2 emulsified in IFA. Arthritis score and incidence were followed for 68 days (all the mice were included in statistics despite onset or not), mice with severe arthritis were killed during the experiment according to ethical permit. The statistical significance given on the top of the symbol of BQ.*Col2*^*266E*^ group represents difference compared with B10Q group at indicating days. Score data were shown as mean ± standard error (same for all the score data in this study) and collected from one experiment. **b** Serum titration of anti-COL2 IgG antibodies was determined at day 35. Data were collected from one experiment. **c** Mice were immunized with rat COL2 emulsified in CFA (Male mice, B10Q: *n* = 6, BQ*.Col2*^*266E*^ homo: *n* = 5, BQ*.Col2*^*266E*^ het: *n* = 3, BQ*.Col2*^*264R*^ homo: *n* = 6, BQ*.Col2*^*264R*^ het: *n* = 3) and killed at day 10 to obtain primed lymph node cells, which were then stimulated with Gal-COL2 peptide to detect the Ag-specific T cells response by ELISpot. Data were collected from one experiment. **d** GIA was induced at day 0 by i.d. injection of GPI peptide emulsified in CFA (Male mice, B10Q: *n* = 10, BQ*.Col2*^*266E*^: *n* = 12). Arthritis severity was followed for 26 days (all the mice were included in statistics despite onset or not). Data were collected from one experiment. Each symbol represents one animal in (**b**) and (**c**). Statistics were done by the Mann–Whitney *U* test, *p < 0.05, **p < 0.01

To determine the tolerance state of BQ*.Col2*^*266E*^ and BQ*.Col2*^*264R*^ mice, the susceptibility to develop arthritis and ex vivo T cell phenotype were checked. We induced CIA in BQ*.Col2*^*266E*^ and BQ*.Col2*^*264R*^ mice by immunization with COL2 at day 0 and boosted at day 35, B10Q mice were used as wild-type control. The results showed that BQ*.Col2*^*266E*^ mice were almost completely resistant to CIA (Fig. 1a) but could still mount some immune response as seen by COL2-specific IgG response at day 35 (Fig. 1b). We further analyzed the T cell-specific response to galactosylated COL2 peptide (Gal-COL2_259–273_) 10 days post immunization through IFN-γ ELISpot. Porcine pepsin was used as positive control since COL2 was prepared with pepsin digestion. Notably, in A^q^-expressing mice, contrary to mice with A^b^ allele, pepsin contamination does not influence arthritis development [27]. The data showed that BQ*.Col2*^*266E*^ mice with either homozygous or heterozygous D266E mutations had much less activated COL2-specific T cells compared with B10Q mice, and pepsin did not affect the results (Fig. 1c). All the evidence suggested that BQ*.Col2*^*266E*^ mice were resistant to the CIA induced by non-self COL2 and did not have detectable autoreactive T cells. Thus, the tighter binding of COL2_259–273_ to A^q^ led to a more efficient presentation of the peptide and induced stronger T cell tolerance.

In contrast, the BQ*.Col2*^*264R*^ mice, in which the COL2 peptide did not share the key endogenous COL2-specific TCR recognition site at position 264, developed arthritis (Fig. 1a). BQ*.Col2*^*264R*^ mice also had comparable anti-COL2 IgG titer and strong ex vivo T cell response to the Gal-COL2_259–273_ peptide which in this case had no indication of tolerance (Fig. 1b, c), suggesting that eliminating the recognition site of TCR could reverse resistance to CIA induced by the D266E mutation. Taken together, both D266E and K264R mutations were as expected functionally dominant, and heterozygous as well as homozygous mice showed identical results.

Meanwhile, to confirm the observed tolerance in the BQ*.Col2*^*266E*^ mice was antigen-specific, we immunized the mice with another self-antigen glucose 6-phosphate isomerase (GPI) peptides, and they were fully susceptible to GPI-induced arthritis (Fig. 1d).

### Additive effect of *Fcgr2b* and *Ncf1* protect against CIA in BQ*.Col2*^*266E*^ mice

After validation of the BQ*.Col2*^*266E*^ strain, we investigated the role of two non-MHC genes that could be of importance for regulating T cell tolerance, i.e., *Fcgr2b* and *Ncf1*. To directly address whether deficiency of *Fcgr2b* or *Ncf1* could break the T cell tolerance to COL2, we crossed B10Q*.Fcgr2b*^*−/−*^ knockout mice and B10Q*.Ncf1*^*m1j/m1j*^ mutated mice with BQ*.Col2*^*266E*^ strain to produce BQ*.Col2*^*266E*^*.Fcgr2b*^*−/−*^ and BQ*.Col2*^*266E*^*.Ncf1*^*m1j/m1j*^ littermates, respectively. These mice were immunized at day 0 with COL2 and boosted at day 35 to induce CIA. B10Q, B10Q*.Fcgr2b*^*−/−*^ and B10Q*.Ncf1 *^*m1j/m1j*^ mice were used as control.

**Fig. 2:**
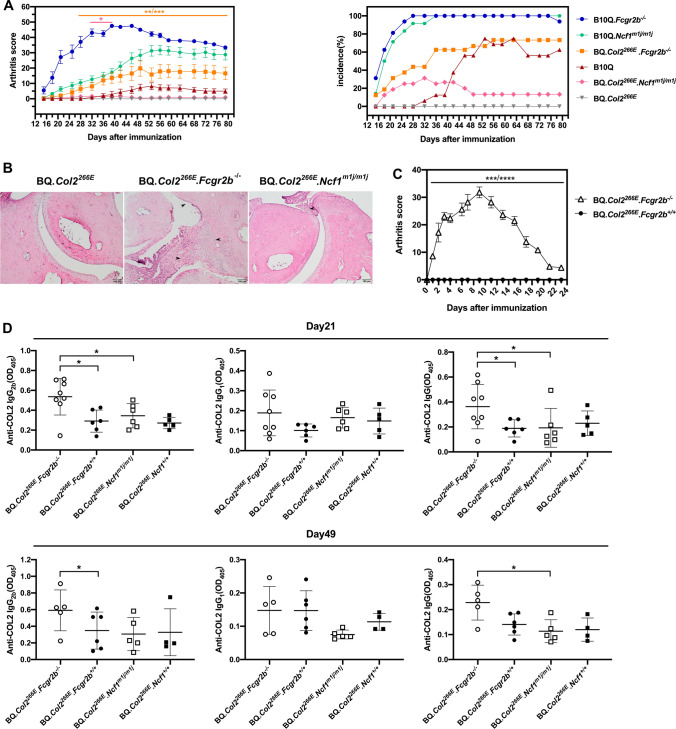
*Fcgr2b* knockout and *Ncf1* mutation reverse resistance to CIA in BQ*.Col2*^*266E*^ mice. *Fcgr2b* knockout mice and *Ncf1*^*m1j/m1j*^ mutated mice were crossed with BQ*.Col2*^*266E*^ strain to produce BQ*.Col2*^*266E*^*. Fcgr2b*^*−/−*^ and BQ*.Col2*^*266E*^*.Ncf1*^*m1j/m1j*^ littermates, respectively. **a** CIA was induced at day 0 by i.d. injection of bovine COL2 emulsified in CFA (Male mice, B10Q*.Fcgr2b*^*−/−*^: *n* = 16, B10Q*.Ncf1*^*m1j/m1j*^: *n* = 12, B10Q: *n* = 17, BQ*.Col2*^*266E*^*. Fcgr2b*^*−/−*^: *n* = 16, BQ*.Col2*^*266E*^*.Ncf1*^*m1j/m1j*^: *n* = 17, BQ*.Col2*^*266E*^: *n* = 14, including BQ*.Col2*^*266E*^*. Fcgr2b*^+*/*+^ and BQ*.Col2*^*266E*^*.Ncf1*^+*/*+^ mice) and mice were boosted at day 35 with bovine COL2 emulsified in IFA. Arthritis score and incidence were followed for 80 days (all the mice were included in statistics despite onset or not), mice with severe arthritis were killed during the experiment according to ethical permit. The upper statistical significance represents difference between BQ*.Col2*^*266E*^*. Fcgr2b*^*−/−*^ group and BQ*.Col2*^*266E*^ group at indicating days, the lower one is between BQ*.Col2*^*266E*^*.Ncf1*^*m1j/m1j*^ and BQ*.Col2*^*266E*^ groups. Data were pooled from two independent experiments with similar results (see details in Table 1). **b** Representative histology images of ankle joints from left hind paws taken at day 90 stained with H&E were showed (scale bar: 100 μm).The arrows from top to bottom in BQ*.Col2*^*266E*^*. Fcgr2b*^*−/−*^ group indicated damaged cartilage, bone erosion and synovial inflammation. The arrow in BQ*.Col2*^*266E*^*.Ncf1*^*m1j/m1j*^ group indicated mild synovitis. **c** CAIA was induced by i.v. injection of pathogenic anti-COL2 antibodies mixture (Male mice, BQ*.Col2*^*266E*^*.Fcgr2b*^*−/−*^: *n* = 5, BQ*.Col2*^*266E*^*.Fcgr2b*^+*/*+^: *n* = 5). Arthritis scores were followed for 23 days. Data were collected from one experiment. **d** Serum levels of anti-COL2 IgG, IgG1, IgG2b antibodies at day 21 and day 49 were shown as OD values (Serum dilution: IgG: 1:6400; IgG_1_:1:1600; IgG_2b_:1:400). Data from different date were separately collected from one experiment. Each symbol represents one animal in (**d**). Statistics were done by the Mann–Whitney *U* test, *p < 0.05, **p < 0.01, ***p < 0.001, ****p < 0.0001

As expected, insertion of *Ncf1*^*m1j/m1j*^ especially *Fcgr2b*^*−/−*^ enhanced the susceptibility to arthritis (Fig. 2a). Importantly, we found that BQ*.Col2*^*266E*^*.Fcgr2b*^*−/−*^ mice developed severe arthritis with high incidence while *Ncf1*^*m1j/m1j*^ mutation allowed the development of arthritis in mice with the D266E mutation but with much milder disease (Fig. 2a) (Table 1). Histology of paw joints taken on day 90 confirmed the scoring data showing severe bone and cartilage destruction and active synovial inflammation in arthritic BQ*.Col2*^*266E*^*.Fcgr2b*^*−/−*^ mice, only synovitis in BQ*.Col2*^*266E*^.*Ncf1*^*m1j/m1j*^ mice and healthy joints in BQ*.Col2*^*266E*^ mice (Fig. 2b). Furthermore, BQ*.Col2*^*266E*^*.Fcgr2b*^*−/−*^ mice showed higher anti-COL2 IgG titers compared with BQ*.Col2*^*266E*^*.Fcgr2b*^+*/*+^ littermates on both day 21 and day 49 (Fig. 2d). We have previously shown that the pathogenic effect of anti-COL2 antibodies is downregulated by FCGR2B*,* and we confirmed this also in BQ*.Col2*^*266E*^ mice, which is much more susceptible to collagen antibody-induced arthritis (CAIA) if deficient in *Fcgr2b* (Fig. 2c). As activation of B cells leading to the production of COL2-specific IgG is T cell-dependent, it argues that T cell tolerance is also affected.

**Table 1:** Arthritis development

Background	*Ncf1*	*Fcgr2b*	Cumulative incidence (%)	Mean maximum score^a^	Mean day of onset^a^
B10Q	+ / +	+ / +	70 (12/17)	14	43
B10Q	+ / +	–/–	100 (16/16)	51	18
B10Q	m1j/m1j	+ / +	100 (12/12)	36	21
BQ*.Col2*^266E^	+ / +	+ / +	0 (0/14)	–	–
BQ*.Col2*^266E^	+ / +	–/–	69 (11/16)	31	27
BQ*.Col2*^266E^	m1j/m1j	+ / +	23 (4/17)	6	22

^a^Including sick animals only

These data together showed that lack of capacity to produce oxidative burst due to the *Ncf1*^*m1j/m1j*^ mutation, or inhibitory signal due to the *Fcgr2b* deficiency could both revert the resistance to CIA in BQ*.Col2*^*266E*^ mice, but to different extents.

### *Fcgr2b*^*−/−*^ and *Ncf1*^*m1j/m1j*^ break T cell tolerance to COL2

**Fig. 3:**
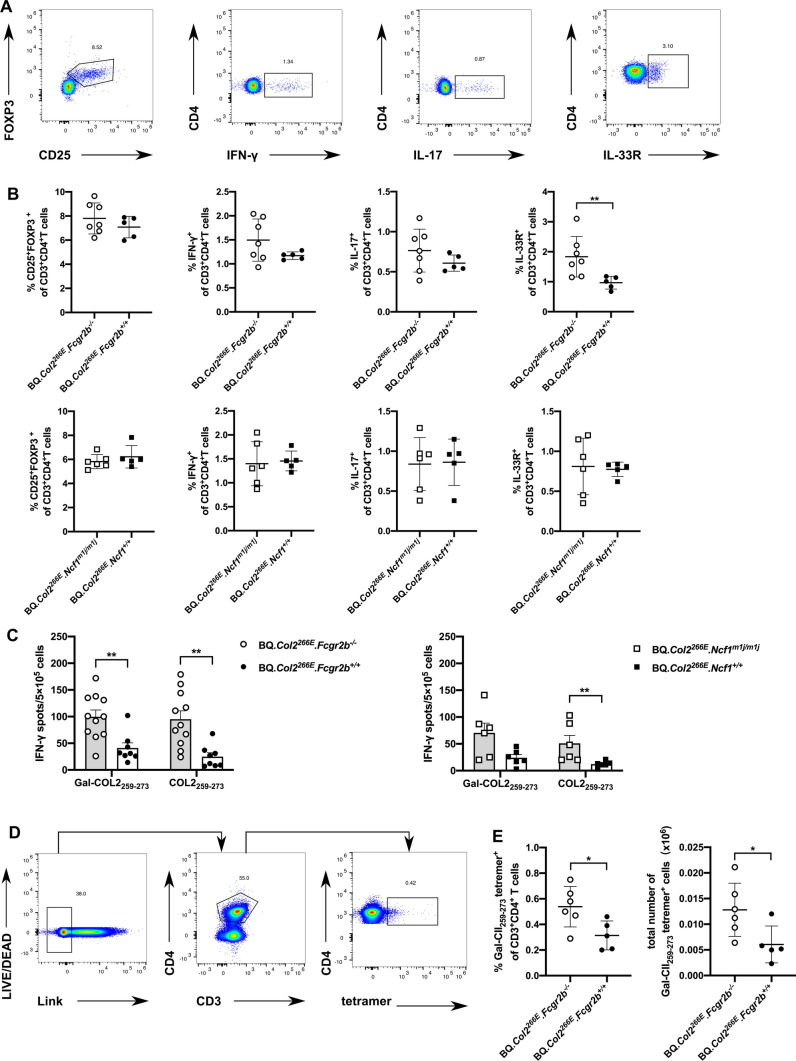
Mice defective in *Fcgr2b* or *Ncf1* allows activation of COL2-specific T cells. Inguinal lymph node cells were obtained at 10 days post immunization with bovine COL2 emulsified in CFA. **a** and **b** Lymph node cells were stained with T cell markers with (for IFN-γ, IL-17 detection) or without PMA stimulation and intracellularly stained for cytokines and FOXP3 to show the T cell sub-populations (Female mice, BQ*.Col2*^*266E*^*. Fcgr2b*^*−/−*^: *n* = 7, BQ*.Col2*^*266E*^*. Fcgr2b*^+*/*+^: *n* = 5, BQ*.Col2*^*266E*^*.Ncf1*^*m1j/m1j*^: *n* = 6, BQ*.Col2*^*266E*^*.Ncf1*^+*/*+^: *n* = 5). Representative FACS gating of Treg cells (CD3^+^CD4^+^CD25^+^FOXP3^+^), CD3^+^CD4^+^IFN-γ^+^ T cells, CD3^+^CD4^+^IL-17^+^ T cells and CD3^+^CD4^+^IL-33R^+^ T cells were showed. The frequencies of each sub-populations among CD3^+^CD4^+^ T cells were showed. For results from BQ*.Col2*^*266E*^*.Fcgr2b*^*−/−*^ and BQ*.Col2*^*266E*^*.Fcgr2b*^+*/*+^ mice, data were collected from one experiment. For results from BQ*.Col2*^*266E*^*.Ncf1*^*m1j/m1j*^ and BQ*.Col2*^*266E*^*.Ncf1*^+*/*+^ mice, data from two experiments with similar results were pooled together. **c** Lymph node cells were stimulated with galactosylated COL2 peptide (Gal-COL2_259–273_) or non-modified COL2 peptide (COL2_259–273_) to detect the Ag-specific T cells by ELISpot (Female mice, BQ*.Col2*^*266E*^*. Fcgr2b*^*−/−*^: *n* = 11, BQ*.Col2*^*266E*^*. Fcgr2b*^+*/*+^: *n* = 8, BQ*.Col2*^*266E*^*.Ncf1*^*m1j/m1j*^: *n* = 6, BQ*.Col2*^*266E*^*.Ncf1*^+*/*+^: *n* = 6). Data from two experiments with similar results were pooled together. **d** Lymph node cells were stained with Gal-COL2_259–273_-specific tetramers (Male mice, BQ*.Col2*^*266E*^*. Fcgr2b*^*−/−*^: *n* = 6, BQ*.Col2*^*266E*^*. Fcgr2b*^+*/*+^: *n* = 5). The FACS gating strategy were showed, Link = CD19, CD11b, MHCII. **e** The frequency of Gal-COL2_259–273_ tetramer^+^ T cells among CD3^+^CD4^+^ T cells were showed, the total Gal-COL2_259–273_ tetramer^+^ T cell number were calculated through the frequency of total lymphocyte times the number of cells read from cell counter. Data were collected from one experiment. Each symbol represents one animal in (**b**), (**c**) and (**e**). Statistics were done by the Mann–Whitney *U* test, *p < 0.05, **p < 0.01

To investigate whether *Fcgr2b*^*−/−*^ deletion and *Ncf1*^*m1j/m1j*^ mutation affect T cell tolerance in the BQ*.Col2*^*266E*^ mice, we firstly addressed a possible shift in the phenotype of CD4^+^ T cells. We collected lymphocytes from draining inguinal lymph nodes 10 days after COL2 immunization in BQ*.Col2*^*266E*^*.Fcgr2b*^*−/−*^ and BQ*.Col2*^*266E*^*.Ncf1*^*m1j/m1j*^ mice with their wild-type littermates, then stained the cells with or without PMA stimulation in vitro to perform flow cytometry. The results showed no difference in the frequency of CD3^+^CD4^+^CD25^+^FOXP3^+^ T cells between groups (Fig. 3a, b), which was also previously found in naïve B6 and B6.*Ncf1*^*m1j/m1j*^ mice [28]. Moreover, we observed a trend of increases in Th1 and Th17 frequencies of CD4^+^ T cells which are strongly related to the development of CIA (Fig. 3a, b), and a clear elevation of IL-33R expressing T cells in BQ*.Col2*^*266E*^*.Fcgr2b*^*−/−*^ mice compared with BQ*.Col2*^*266E*^*.Fcgr2b*^+*/*+^ littermates (Fig. 3a, b). IL-33 has previously been reported its pathological roles in RA [29] and activation of IL33R^+^ T cells has been shown to increase the incidence of adjuvant-free CIA driven by Gal-COL2-specific T cells [30].

We also analyzed the antigen-specific T cells response in the primed lymph node cells from BQ*.Col2*^*266E*^*.Fcgr2b*^*−/−*^ and BQ*.Col2*^*266E*^*.Ncf1*^*m1j/m1j*^ mice 10 days after immunization with COL2. These cells were stimulated with non-modified COL2 peptide (COL2_259–273_) or Gal-COL2_259–273_ peptide and analyzed with IFN-γ ELISpot assays. The results showed that BQ*.Col2*^*266E*^*.Fcgr2b*^*−/−*^ mice had greatly increased autoreactive T cell responses to both non-modified and Gal-COL2 peptides (Fig. 3c), suggesting *Fcgr2b* could be important for T cell tolerance considering that tolerance to non-modified COL2 peptide is established in the thymus [18]. The BQ*.Col2*^*266E*^*.Ncf1*^*m1j/m1j*^ also showed an increased T cell response towards non-modified COL2 peptide compared with littermates, which is consistent with previous findings with MMC mice [31] (Fig. 3c). To further confirm the finding of increased autoreactive T cells, we performed Gal-COL2_259–273_-specific tetramers staining in lymph node cells from BQ*.Col2*^*266E*^*.Fcgr2b*^*−/−*^ and BQ*.Col2*^*266E*^*.Fcgr2b*^+*/*+^ littermates 10 days after COL2 immunization. The results showed that both the frequency and total number of Gal-COL2-specific T cells were all elevated in BQ*.Col2*^*266E*^*.Fcgr2b*^*−/−*^ mice (Fig. 3d, e). Thus, both *Fcgr2b*^*−/−*^ and *Ncf1*^*m1j/m1j*^ could break T cell tolerance to COL2 and increase the autoreactive T cells.

### *Fcgr2b *and *Ncf1* regulate tolerance through different mechanisms

**Fig. 4:**
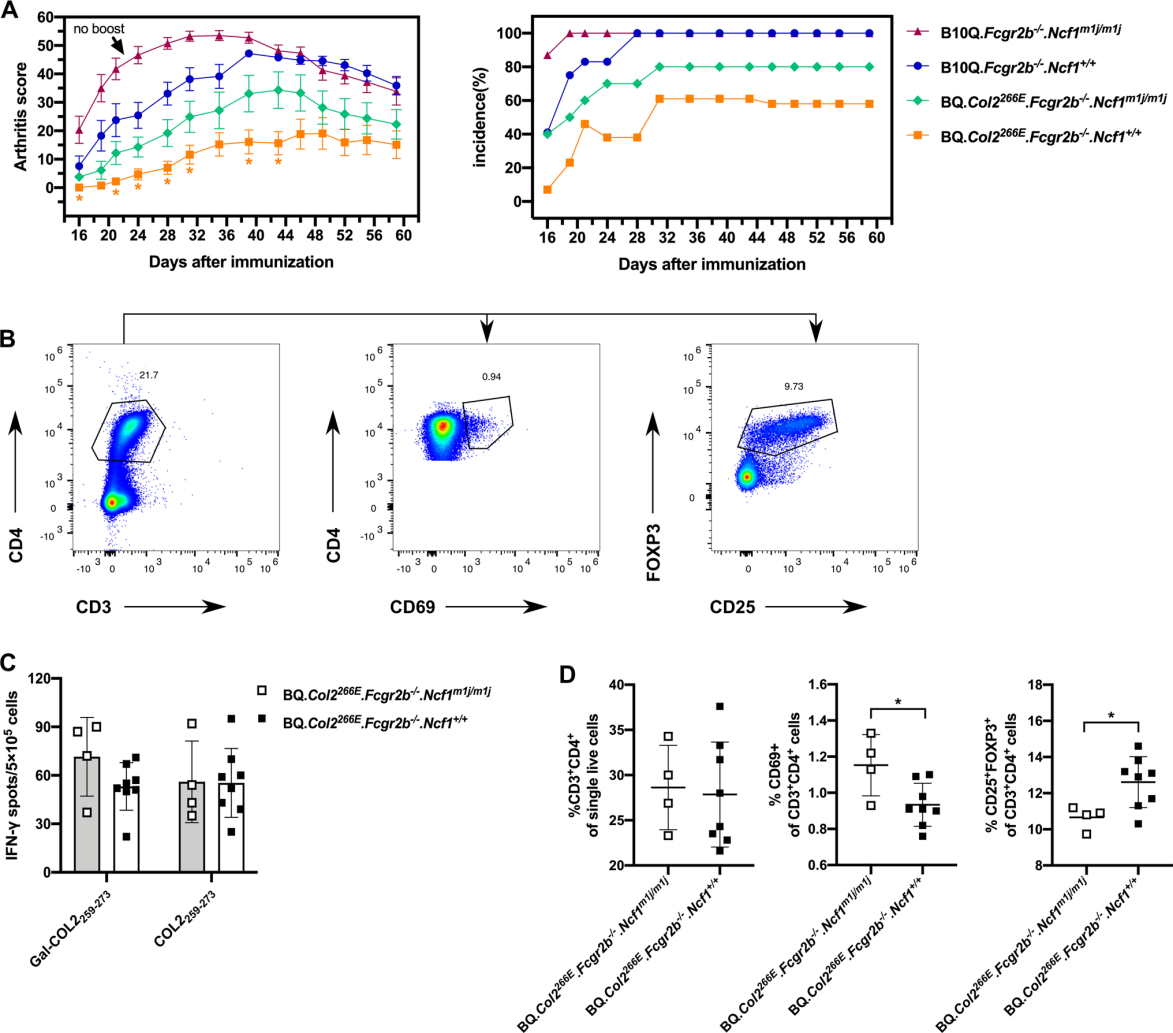
Both *Fcgr2b* and *Ncf1* deficiency contributed to the enhanced arthritis susceptibility. B10Q*.Fcgr2b*^*−/−*^*.Ncf1*^*m1j/m1j*^ and BQ*.Col2*^*266E*^*.Fcgr2b*^*−/−*^*.Ncf1*^*m1j/m1j*^ were developed to address the interaction between *Fcgr2b* and *Ncf1*. **a** Mice from indicated groups were i.d. injected with bovine COL2 emulsified in CFA and were boosted at day 35 with bovine COL2 emulsified in IFA (except for B10Q*.Fcgr2b*^*−/−*^*.Ncf1*^*m1j/m1j*^ group). Arthritis score and incidence were followed for 60 days (all the mice were included in statistics despite onset or not), mice with severe arthritis were killed during the experiment according to ethical permit. (Male mice, B10Q*.Fcgr2b*^*−/−*^*.Ncf1*^*m1j/m1j*^: *n* = 8, B10Q*.Fcgr2b*^*−/−*^*.Ncf1*^+*/*+^: *n* = 12, BQ*.Col2*^*266E*^*.Fcgr2b*^*−/−*^*. Ncf1*^*m1j/m1j*^: *n* = 10, BQ*.Col2*^*266E*^*.Fcgr2b*^*−/−*^*. Ncf1*^+*/*+^: *n* = 13).Data were collected from one experiment. **b** and **d** Draining lymph node cells were obtained at 10 days post bovine COL2 immunization (Female mice, BQ*.Col2*^*266E*^*.Fcgr2b*^*−/−*^*. Ncf1*^*m1j/m1j*^: *n* = 4, BQ*.Col2*^*266E*^*.Fcgr2b*^*−/−*^*. Ncf1*^+*/*+^: *n* = 8),CD3^+^CD4^+^ CD69^+^ T cells and CD3^+^CD4^+^CD25^+^FOXP3^+^ Treg cells were detected by FACS. The represent FACS gating and the frequency of each population were showed. Data were collected from one experiment. **c** Draining lymph node cells were obtained at 10 days post bovine COL2 immunization and stimulated with galactosylated COL2 peptide (Gal-COL2_259–273_) or non-modified COL2 peptide (COL2_259–273_) (Female mice, BQ*.Col2*^*266E*^*.Fcgr2b*^*−/−*^*. Ncf1*^*m1j/m1j*^: *n* = 4, BQ*.Col2*^*266E*^*.Fcgr2b*^*−/−*^*. Ncf1*^+*/*+^: *n* = 8). The COL2-specific T cells were detected by IFN-γ ELISpot. Data were collected from one experiment. Each symbol represents one animal in (**c**) and (**d**). Statistics were done by the Mann–Whitney *U* test, *p < 0.05

As both *Fcgr2b* and *Ncf1* protected against the breach of T cell tolerance and development of arthritis, we next addressed the possibility that they operate in the same pathway, i.e., that they epistatically interact. Therefore, we made new cross-breeding on the BQ*.Col2*^*266E*^ or B10Q background inducing deficiency of both *Fcgr2b* and *Ncf1* and immunized them with COL2 together with control groups, then recorded the development of arthritis. The results showed B10Q*. Fcgr2b*^*−/−*^*.Ncf1*^*m1j/m1j*^ developed the most severe arthritis with early onset and 100% incidence, compared with other strains (Fig. 4a). Thus, both *Ncf1* and *Fcgr2b* deficiency contributed to the arthritis susceptibility. Similarly, BQ*.Col2*^*266E*^*. Fcgr2b*^*−/−*^*.Ncf1*^*m1j/m1j*^ mice, with the D266E mutation, had more severe disease than BQ*.Col2*^*266E*^*. Fcgr2b*^*−/−*^*.Ncf1*^+*/*+^ littermates (Fig. 4a). The interaction between *Fcgr2b* and *Ncf1* was additive, indicating that the pathways whereby they influence arthritis are mainly independent on each other. It does not exclude an epistatic interaction involving some part of the effect that could be hidden by other effects, for example, FcR stimulated by immune complexes (IC) could activate the NOX2 complexes inducing a substantial release of ROS. And we showed that *Fcgr2b* deficiency could lead to elevated ROS production induced by IC in DCs in vitro (suppl Fig. 1).

We proceeded to perform a T cell recall assay and checked the phenotype of T cells from draining lymph nodes of BQ*.Col2*^*266E*^*.Fcgr2b*^*−/−*^ mice, with different *Ncf1* alleles, 10 days post immunization and found interesting differences. Although the numbers of IFNγ-expressing COL2-specific T cells did not differ significantly (Fig. 4c), there was a relative expansion of activated CD4^+^CD69^+^ T cells and a reduction of FOXP3^+^ Tregs in mice with deficient *Ncf1* (Fig. 4b, d), indicating that *Ncf1* might influence T cell tolerance through Tregs generation.

### Different roles of FCGR2B and NCF1 on antigen-presenting cells in thymus

**Fig. 5:**
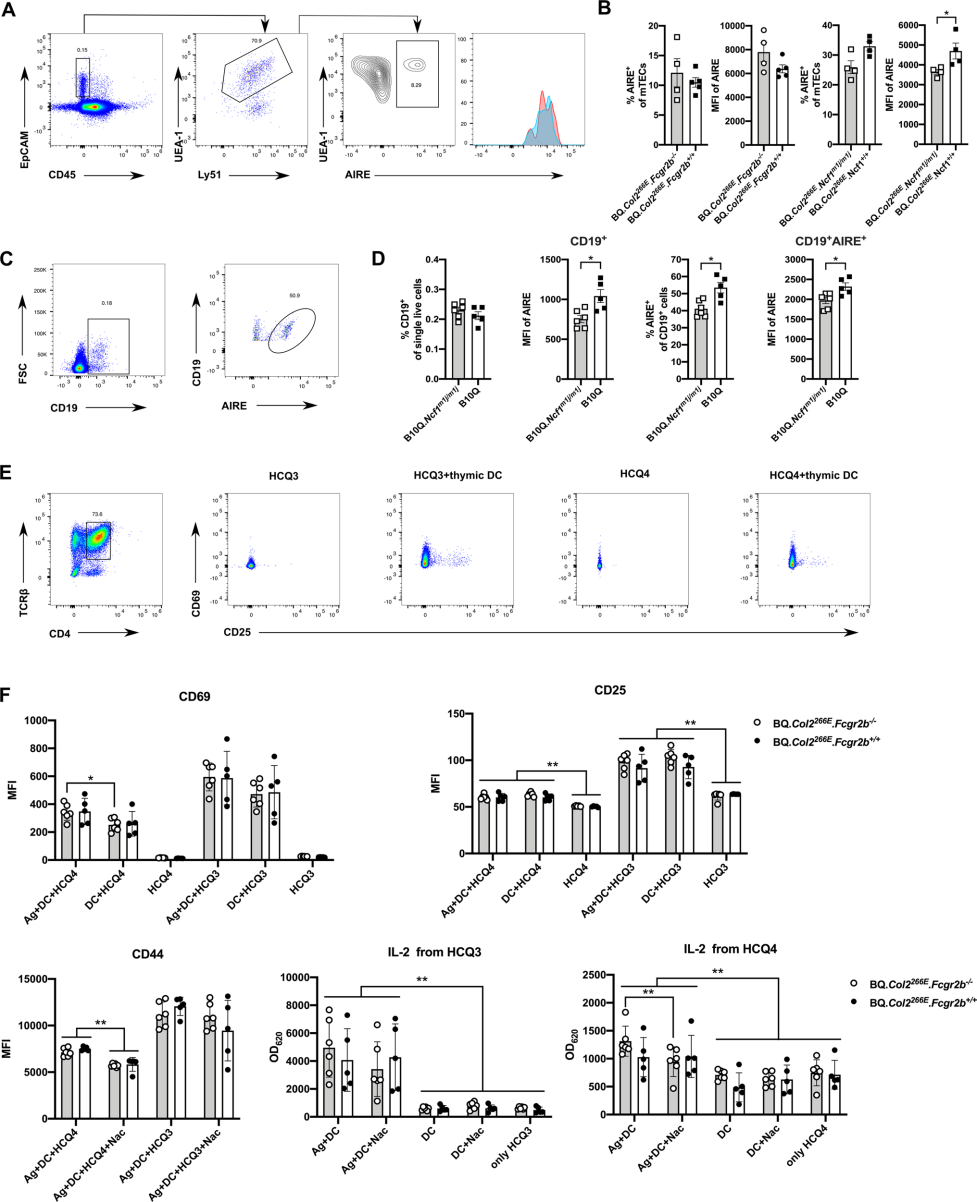
The role of *Fcgr2b* or *Ncf1* on antigen-presenting cells in thymus. **a** Thymi were obtained from naïve BQ*.Col2*^*266E*^*.Fcgr2b*^*−/−*^ and BQ*.Col2*^*266E*^*.Ncf1*^*m1j/m1j*^ mice together with their littermates then digested and stained for AIRE. Representative FACS images of mTECs gating (CD45^−^EpCAM^+^UEA-1^high^) and histogram of AIRE expression were shown (red: BQ*.Col2*^*266E*^*.Fcgr2b*^*−/−*^, blue: BQ*.Col2*^*266E*^*.Fcgr2b*^+*/*+^). **b** The frequency of AIRE^+^ mTECs among total mTECs and AIRE expression in AIRE^+^ mTECs (shown as mean fluorescence intensity (MFI)) from BQ*.Col2*^*266E*^*.Fcgr2b*^*−/−*^ and BQ*.Col2*^*266E*^*.Ncf1*^*m1j/m1j*^ mice and their littermates were showed. (Male mice: BQ*.Col2*^*266E*^*. Fcgr2b*^*−/−*^: *n* = 4, BQ*.Col2*^*266E*^*. Fcgr2b*^+*/*+^: *n* = 5, Female mice: BQ*.Col2*^*266E*^*.Ncf1*^*m1j/m1j*^: *n* = 4, BQ*.Col2*^*266E*^*.Ncf1*^+*/*+^: *n* = 4). Data were collected separately for BQ*.Col2*^*266E*^*.Fcgr2b* littermates and BQ*.Col2*^*266E*^*.Ncf1* littermates, both from one experiment. **c** and **d** Thymi were obtained from naïve B10Q and B10Q*.Ncf1 *^*m1j/m1j*^ mice and stained for AIRE (Male mice: B10Q*.Ncf1 *^*m1j/m1j*^:*n* = 6, B10Q:*n* = 5). Representative FACS images of thymic B cells (CD45^+^CD19^+^) gating and AIRE^+^ B cells (CD45^+^CD19^+^AIRE^+^) gating were shown. Thymic B cells frequency of single live cells and AIRE^+^ B cells frequency of thymic B cells were shown. AIRE expressions of these populations were also shown as MFI, respectively. Data were collected from one experiment. **e** Representative FACS images of CD4^+^TCRb^+^ T hybridoma cells gating and CD69 or CD25 expression on hybridoma T cells with or without incubation with thymic DCs were shown, no extra antigen was added. **f** HCQ3 and HCQ4 hybridoma T cells were incubated with or without thymic DCs in the presence of COL2 molecules (Antigen) or no antigen for 15 h, 5 mM *N*-*acetyl*-*L*-*cysteine* (Nac) were added in some groups to remove ROS. After incubation, the supernatant medium was measured for IL-2 by ELISA, the results were shown as OD values (OD_620_). The rest part was stained for CD69/CD25/CD44, then detected by FACS, the results were shown as MFI, respectively (Female mice: BQ*.Col2*^*266E*^*. Fcgr2b*^*−/−*^: n = 6, BQ*.Col2*^*266E*^*. Fcgr2b*^+*/*+^: *n* = 5). Data were collected from one experiment. Each symbol represents one animal in (**b**), (**d**) and (**f**). Statistics were done by the Mann–Whitney *U* test, *p < 0.05, **p < 0.01

Previous studies have shown that medullary thymic epithelial cells (mTECs), as well as recirculating DCs express and present a variety of tissue-restricted antigens (TRAs) including COL2 peptide in a transcription factor AIRE-dependent way [18, 32], which is the key step for negative selection of COL2-specific T cells in the thymus. To investigate whether FCGR2B or NCF1 could influence central T cell tolerance by affecting AIRE, we first checked the expression of AIRE on mTECs and other FCGR2B-expressing APCs in the thymus. We isolated thymi from naïve BQ*.Col2*^*266E*^*.Fcgr2b*^*−/−*^ mice and BQ*.Col2*^*266E*^*.Fcgr2b*^+*/*+^ littermates, then differentiated mTECs (CD45^−^EpCAM^+^UEA-1^high^) as previously described [25] (Fig. 5a) as well as B cells (CD45^+^CD19^+^), macrophages (CD45^+^CD11b^+^F4/80^+^), neutrophils (CD45^+^CD11b^+^Ly6G^+^), DCs (CD45^+^Link^−^CD11c^+^) by flow cytometry (fig. S2a). The results showed that FCGR2B was not expressed on mTECs (Fig. S2b). The expression of AIRE in mTECs, B cells and other FCGR2B-expressing cells in the thymus were not affected by FCGR2B deficiency (Figs. 5b, S2a).

NCF1 on the other hand was expressed on mTECs and other thymus APCs (Fig.S2c), suggesting that NCF1 deficiency might affect the function of mTECs. Therefore, we went on to check the AIRE expression in BQ*.Col2*^*266E*^*.Ncf1*^*m1j/m1j*^ mice and compared with BQ*.Col2*^*266E*^*.Ncf1*^+*/*+^ littermates. The results showed that the *Ncf1*^*m1j/m1j*^ mutation could to some extent decrease the expression of AIRE in mTECs (Fig. 5b) indicating the possibility of an impaired TRAs expression and presentation. Interestingly, besides BQ*.Col2*^*266E*^ background mice, we also found that the *Ncf1* mutation could decrease the AIRE expression in thymus B cells in B10Q*.Ncf1*^*m1j/m1j*^ mice compared with wild-type mice (Fig. 5c, d), suggesting the possible range of *Ncf1* effects.

Because FCGR2B was found not to be expressed on mTECs, we hypothesized that it might affect central tolerance through other FCGR2B-expressing APCs in the thymus. DCs express FCGR2B and have been shown to be an important APC type in central T cell tolerance induction [33, 34]. To determine whether thymus DCs could pick up the COL2 peptide in vivo and present it to autoreactive thymocytes, we used two kinds of T cell hybridomas producing IL-2 after stimulation—HCQ3 cells which are specific for the Gal-COL2 peptide and HCQ4 cells which are specific for non-modified COL2 peptide [26]. We isolated single cells from thymi obtained from naïve BQ*.Col2*^*266E*^*.Fcgr2b*^*−/−*^ mice and BQ*.Col2*^*266E*^*.Fcgr2b*^+*/*+^ littermates and sorted DCs by magnetic separation using ultra-pure CD11c microbeads. We incubated the thymic DCs with either HCQ3 or HCQ4 cells with or without corresponding antigens in the medium, then detected the T cells activation markers CD25 and CD69 on T cell hybridomas by flow cytometry, and IL-2 in the supernatants was measured by ELISA. The results showed that both T cells responded even without extra added antigens, indicating that the thymus DCs were loaded with both non-modified and Gal-COL2 peptides, which could activate COL2-specific T cell hybridomas (Fig. 5e), confirming previous data on recirculating thymic DC[18]. However, thymic DCs from BQ*.Col2*^*266E*^*. Fcgr2b*^*−/−*^ mice had an unaltered ability to activate HCQ3 and HCQ4 T cells compared with BQ*.Col2*^*266E*^*.Fcgr2b*^+*/*+^ mice (Fig. 5f). Still, the activation function of the DCs is redox-regulated as we noticed that removing ROS by adding 5 mM *N*-*acetyl*-*L*-*cysteine* (Nac) decreased the expression of activation marker CD44 and IL-2 secretion of the hybridoma T cells (Fig. 5f)*.*

Taken together, both *Fcgr2b* and *Ncf1* regulate immune tolerance but through different mechanisms. Deficiency of *Ncf1* decreased the numbers of Tregs, an effect that may depend on antigen-presenting cells (mTECs, B cells and DCs) in the thymus, which was likely to be associated with decreased AIRE expression.

## Discussion

In both mice and humans, the T cell response to COL2 is directed to the same immunodominant target—the COL2_259–273_ peptide binding Aq in mice and DR*0401 in humans [13], which is also the key immune target in CIA. In this study, we showed that a mutation of COL2 in mice changing the aspartic acid (D) to a glutamic acid (E) at position 266 (as in humans), allowing a stronger binding to the MHCII molecules, leads to profound T cell tolerance and decreased susceptibility to arthritis. FCGR2B, as well as NCF1, could break the T cell tolerance although through different mechanisms. NCF1 is likely to be associated with the control of thymus-derived Treg development.

This study provides a mouse model for the human autoreactive T cell response to COL2 and allows studies of T cell tolerance regulation. Such studies are not possible in the classical CIA model, which activates heterogenous COL2-reactive T cells that do not recognize the endogenous COL2 peptide in vivo. Also, we clearly demonstrated that by using the BQ*.Col2*^*264R*^ mice in which the major T cell recognition site is eliminated through mutation of the lysine at position 264, allowing a pure non-self T cell response, resulted in severe arthritis. We have earlier used a transgenic model which express mutated mouse COL2 [17] but with unphysiological expression, since the endogenous mouse COL2 is also expressed, and heterotrimeric molecules will be formed. In comparison, the transgenic model showed less pronounced protection against arthritis together with the *Ncf1* mutation [31].

*Ncf1* was first positionally cloned from rats with autoimmune arthritis [7] and later positioned both as a copy number variation (CNV) and disease causative SNP (rs201802880, or NCF1–339) in humans [8–10]. The *Ncf1*^*m1j/m1j*^ mutations decreasing NCF1 function, leading to a diminished induction of ROS, are causative alleles for both arthritis and lupus [7–10, 20, 21, 35]. The downstream mechanisms explaining the exaggerated activation of T cells are however likely to be complex, involving not only oxidation of antigenic peptides during processing and changing the activity of the APCs but also directly affecting the interacting T cells by changing T cell surface redox level or possibly triggering differentiation to Tregs [36–38]. We have previously proposed the possibility that ROS from APCs may operate as an immunologic transmitter to regulate T cells auto-reactivity [39], which could affect TCR signaling through lipid rafts in the plasma membrane that are believed to be regulated by redox signaling [40]. Whether T cell selection in the thymus is oxidatively regulated is still not known, but it has been reported that single positive CD4^+^ thymocytes developed an increased level of ROS during maturation [41]. In our study, we reported that the *Ncf1*^*m1j*^ mutation could to some extent lower the AIRE expression in mTECs, thus affecting the expression and presentation of TRAs which could reduce the efficiency of T cell-negative selection and promote the release of potential autoreactive T cells to the periphery [42]. Interestingly, *Aire* knockout can also reduce the ROS level in CD4^+^ SP thymocytes in B6 mice [41]. Using the new BQ*.Col2*^*266E*^ mice, we showed that *Ncf1* mutation leads to decreased Tregs. COL2 peptide is presented during T cell selection in the thymus and the observed downregulation of AIRE in mTECs and B cells might explain the observed reduction of Tregs. These results add other possibilities on how NCF1 regulates the immune system.

As the only inhibitory IgG Fc receptor, FCGR2B could counterbalance the activation signals by co-ligation with activating FcRs or BCR through immune complexes to exert its diverse regulatory functions in innate and adaptive immunity. Deficiency of *Fcgr2b* is associated with several autoimmune diseases including SLE, RA, anti-glomerular basement membrane (GBM) disease, and multiple sclerosis (MS) [43]. Mouse strains with targeted *Fcgr2b* deletions have been extensively studied on different models resulting in diverse reports regarding its role in autoimmunity [44, 45]. Briefly, in autoimmune arthritis models, deficiency of *Fcgr2b* could increase the susceptibility mainly through autoantibody production, and the regulation of the DCs function as well as peripheral B cell tolerance [46, 47]. Although studies have reported various pathways for *Fcgr2b* regulating the T cell function [48, 49], not much were specifically focused on its role in T cell tolerance. The difficulties to sort out its role could partly be due to linked effects with neighboring genes, including other Fc-receptors. Notably, in this study, we used a congenic fragment originally from the 129 strain containing a targeted *Fcgr2b* deletion, which means other genes besides *Fcgr2b* could contribute to the results. In a forward genetic cloning study, we identified that both *FcgR3* and *Fcgr2b* alleles within the same haplotype, both contribute and operate in an additive way [22].

We now showed that in a controlled autoimmune setting, deficiency of *Fcgr2b* is associated with activation of autoreactive T cells and enhancement of CIA. It is known that activation of FcR induce a ROS response through activation of the NOX2 complex and we initially hypothesized that *Ncf1* and *Fcgr2b* genes could interact. However, we have no evidence for this, instead the major mechanism by which these two genes control the induction of T cell tolerance and enhance arthritis seem to be different.

We conclude that for the first time a new human-mimicking model for RA is described, in which an adequate autoreactive MHCII-restricted T cell response to COL2 can be followed. Both *Ncf1* and *Fcgr2b* locus are important to maintain T cell tolerance, providing insights for more possibilities of their regulatory functions.

## Conflict of interest

The authors have no relevant financial or non-financial interests to disclose.

## Ethical approval

The animal genotyping and experimental protocols used in this study were approved by Stockholm regional animal ethics committee, Sweden (12923-2018, N35/16).

## Supplementary Information

Below is the link to the electronic supplementary material.

Supplementary file1 (DOCX 756 KB)

## Data Availability

The datasets generated and analyzed during the current study are available from the corresponding author on reasonable request.
