# Supplementary material for: Two major genes associated with autoimmune arthritis, Ncf1 and Fcgr2b, additively protect mice by strengthening T cell tolerance
Source: Cell Mol Life Sci. 2022 Aug 14;79(9):482. doi: 10.1007/s00018-022-04501-0 (PMC9375767; doi:10.1007/s00018-022-04501-0)
Supplement: Supplementary file 1 — Supplementary file1 (DOCX 756 KB) [file 18_2022_4501_MOESM1_ESM.docx]

**Figure S1**

**
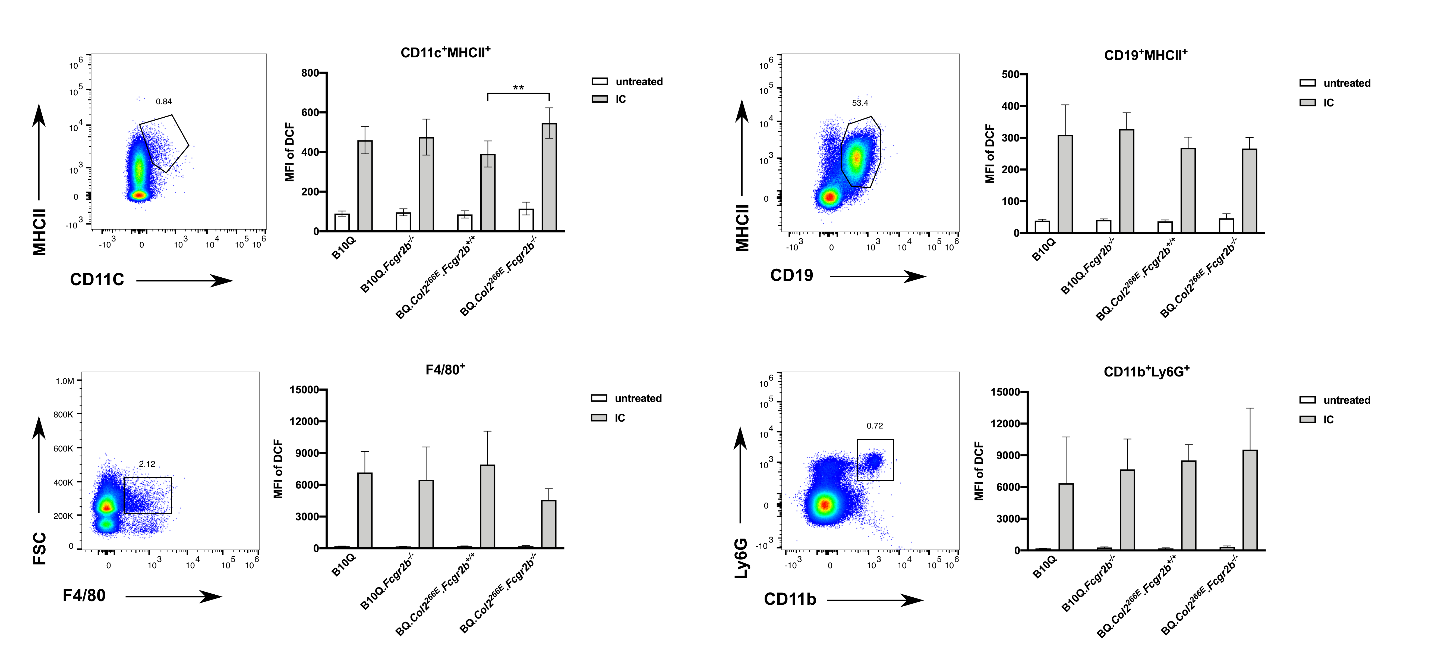
**

**Figure S1 Immunocomplex induced ROS production**

Spleen cells were obtained from naïve indicated mice to detect the ROS production induced by immunocomplex conjugated with DCF from FCGR2B-expressing cells: DCs (CD11c^+^MHCII^+^), B cells (CD19^+^MHCII^+^), macrophages (F4/80^+^), neutrophils (CD11b^+^Ly6G^+^).(Female mice, B10Q:n=4; B10Q*.Fcgr2b^-/-^*:n=5; BQ*.Col2^266E^*. *Fcgr2b*^+/+^:n=5; BQ*.Col2^266E^*. *Fcgr2b^-/-^*:n=5). Representative FACS images of gating and mean fluorescence intensity (MFI) of DCF were shown. Data were collected from one experiment. Statistics were done by the Mann-Whitney U test, **p < 0.01.

**Figure S2**

**
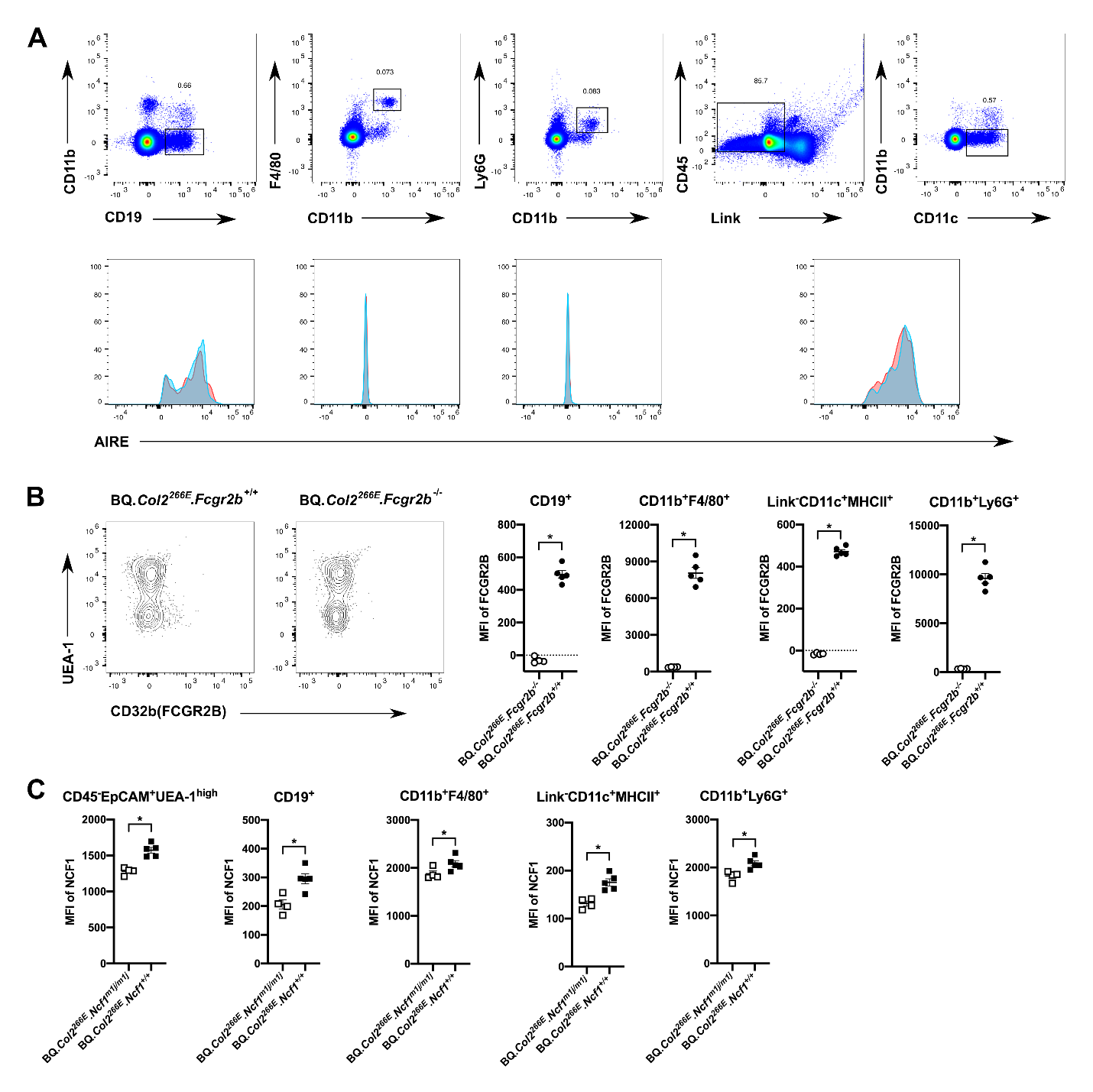
**

**Figure S2 Expression of AIRE, FCGR2B and NCF1 on thymic APCs**

(a) Thymi were obtained from naïve BQ*.Col2^266E^.Fcgr2b^-/-^*and BQ*.Col2^266E^.Fcgr2b^+/+^* mice (Male mice: BQ*.Col2^266E^. Fcgr2b^-/-^*: n=4, BQ*.Col2^266E^. Fcgr2b^+/+^*: n=5) then digested and stained for AIRE. Representative gating of FCGR2B-expressing cells from B cells (CD45^+^CD19^+^), macrophages (CD45^+^CD11b^+^F4/80^+^), neutrophils (CD45^+^CD11b^+^Ly6G^+^) DCs (CD45^+^Link^-^CD11c^+^), and histogram of AIRE expression were shown (red: BQ*.Col2^266E^.Fcgr2b^-/-^*, blue: BQ*.Col2^266E^.Fcgr2b^+/+^*). Link= CD4, CD8a, TCRb, TCRgd, CD11b, NK1.1, Gr1, Ter-119. Data were collected from one experiment. (b) Thymic APCs from naïve BQ*.Col2^266E^.Fcgr2b^-/-^*and BQ*.Col2^266E^.Fcgr2b^+/+^* (Female mice: BQ*.Col2^266E^. Fcgr2b^-/-^*: n=4, BQ*.Col2^266E^. Fcgr2b^+/+^*: n=5) were stained for CD32b (FCGR2B). Representative FACS images of mTECs (UEA-1^high^) and cTECs (UEA-1^low^) were shown. CD32b expression for B cells (CD45^+^CD19^+^), macrophages (CD45^+^CD11b^+^F4/80^+^), neutrophils (CD45^+^CD11b^+^Ly6G^+^) DCs (CD45^+^Link^-^CD11c^+^MHCII^+^) were shown as MFI. Data were collected from one experiment. (c) Thymi were obtained from naïve BQ*.Col2^266E^.Ncf1^m1j/m1j^* and BQ*.Col2^266E^.Ncf1^+/+^*littermates (Female mice: BQ*.Col2^266E^.Ncf1^m1j/m1j^*: n=4, BQ*.Col2^266E^.Ncf1^+/+^*: n=5) and stained for NCF1 in mTECs and other cell types mentioned above. The NCF1 expression were shown as MFI. Data were collected from one experiment. Each symbol represents one animal in (b) and (c) Statistics were done by the Mann-Whitney U test, *p < 0.05.
